# Editorial: The relationship between exercise addiction, social media use, and mental health

**DOI:** 10.3389/fpsyt.2024.1459166

**Published:** 2024-08-01

**Authors:** Annagiulia Di Trana, Alessandro Di Giorgi, Roberta Tittarelli

**Affiliations:** ^1^ National Centre on Addiction and Doping, Istituto Superiore di Sanità, Rome, Italy; ^2^ Department of Biomedical Science and Public Health, University Politecnica delle Marche, Ancona, Italy; ^3^ Department of Biomedicine and Prevention, University of Rome Tor Vergata, Rome, Italy

**Keywords:** exercise addiction, Fear of Missing Out (FoMO), social media use disorder, mental health, behavior disorders

Exercise addiction (EA) is a dysfunctional behavior characterized by an obsessive engagement in physical exercise, which may lead to unhealthy conditions and negative physical, psychological, and social consequences (1). Whereas regular physical activity positively influences lifestyle, several studies have recently discussed the possible correlation between excessive involvement in physical exercise and mental health disorders, such as compulsive behavior. In this context, social media exacerbate the pressure on fragile individuals to achieve an ideal body shape. Indeed, the typical manifestations of EA are compulsive exercise, the absence of rest sessions during the exercise, and the disregard for injuries (2). The consequent mental health disorders are represented by negative emotions, prolonged exercise time, insomnia, and a sense of deprivation when it is not possible to exert physical activity. However, an official definition of EA and the precise profiling of individuals who suffer from EA are still lacking due to the scarce scientific evidence in the literature.

To this concern, this research topic aimed to collect the most recent studies focusing on the description of the adverse effects of EA on general and mental health to promptly distinguish problematic behavioral addiction from non-pathological excessive exercise. Furthermore, an important focus has been provided on the correlation between EA and mental health disorders and the role that social media use (SMU) may play in this condition.

The problematic consequences of SMU in correlation to fear of missing out (FoMO) was the focus of Ye et al. In particular, they investigated the effect of FoMO on learning burnout in medical students, which may significantly affect their academic performance and professional growth. Firstly, the authors administered a survey to 352 medical students to assess their anxiety levels correlated to missing out on social activities and learning burnout, according to the FoMO Scale (FoMOS) and the Learning Burnout Scale (LBS), respectively. The results showed a positive correlation between FoMO and learning burnout. Next, the authors investigated how FoMO influences academic burnout; for this purpose, 3,085 college students completed an online survey. Three additional scales were applied for the evaluation of results, namely, the Chinese version of the Smartphone Addiction Scale (SAS), the Pittsburg Sleep Quality Index (PSQI), and the Five Facet Mindfulness Questionnaire (FFMQ). Statistical analyses revealed positive correlations between FoMO and smartphone addiction, FoMO and learning burnout, smartphone addiction and learning burnout, sleep quality and FoMO, sleep quality and smartphone addiction, and sleep quality and learning burnout. Considering these preliminary results, Ye et al. recruited 30 medical students for a 4-week mindfulness intervention. Participants were required to join a mindfulness training group course 1 h per week for 4 weeks. The scales adopted for the first study step were applied, and the results showed the validity of mindfulness training. In particular, the smartphone addiction and learning burnout scores significantly decreased after the training period; however, the FoMO score did not significantly decrease.

The effects of empathy on the bidirectional relationships between problematic smartphone use (PSU) and aggression among secondary school students were studied by Wu et al. by administering a questionnaire to 2,469 students. For this purpose, the Basic Empathy Scale, the Buss-Warren Aggression Questionnaire, and the Mobile Phone Addiction Index (MPAI) were applied to evaluate the students’ empathy level, aggressiveness, and PSU, respectively. Results showed several edges correlating the symptoms of aggression and PSU, suggesting that these disorders may bidirectionally interact. Moreover, the strongest correlation was observed between “hostility” and “withdrawal/escape.” In addition, the highest expected influence (EI) was obtained by “anger” in both affective and cognitive moderate network models. Interestingly, “productive loss” and “physical aggression” presented different conditional effects depending on the levels of affective empathy. In particular, a positive bidirectional relationship was observed between these behaviors and lower levels of affective empathy, while this bidirectional association turned negative at higher levels.

Wu et al. investigated the mediating role of resilience and interaction anxiousness in the effects of physical activity on mobile phone addiction (MPA). For this purpose, 590 college students were requested to complete a psychosocial battery, evaluated by applying the International Physical Activity Questionnaire - Short Form (IPAQ-SF), the Connor-Davidson Resilience Scale (CD-RISC), the Interaction Anxiousness Scale (IAS), and the MPAI. Results revealed that higher levels of physical activity were associated with lower levels of phone addiction, indicating a negative correlation between these disorders. Furthermore, students with higher resilience demonstrated a positive effect of physical activity on reducing MPA. Thus, resilience played a crucial role in moderating this behavioral disorder, while interaction anxiousness had the opposite effect; indeed, students with higher levels of interaction anxiousness presented a weaker positive effect of physical activity on reducing MPA. Another important result is the chain-mediating effect that physical activity exerts on MPA. Specifically, physical exercise may enhance resilience, reducing anxiousness and decreasing MPA behavior.

A comprehensive literature search was performed by Minutillo et al., who presented a review on the correlation between the use of social media, EA, and personality traits (such as perfectionism, body dissatisfaction, and depression). The search followed the PRISMA guidelines and included 15 articles, which highlighted that EA is correlated to the abovementioned personality disorders. Contrastingly, a controversial relationship was noticed between SMU and EA. Indeed, some studies reported that passive SMU was correlated to a low rate of physical activity practice (minimum of 1 h), while active SMU was associated with a higher probability of exercise activity. However, other authors observed that the latter was not linked to a passive or active use of social networks.

In conclusion, this Research Topic highlighted the implications of EA on both general and mental health, outlining some important correlations between personality traits and the possibility of developing this understudied behavioral disorder. All the studies showed the importance of exercise addiction and the need for prompt action, highlighting some concurrent behaviors. Indeed, the reported contributing factors need to be evaluated by healthcare professionals to better address and mitigate the adverse effects of this behavioral addiction.

## Author contributions

ADT: Writing – original draft, Writing – review & editing. ADG: Writing – original draft, Writing – review & editing. RT: Writing – original draft, Writing – review & editing.
